# Accurate Localization of the Integration Sites of Two Genomic Islands at Single-Nucleotide Resolution in the Genome of *Bacillus cereus* ATCC 10987

**DOI:** 10.1155/2008/451930

**Published:** 2008-04-17

**Authors:** Ren Zhang, Chun-Ting Zhang

**Affiliations:** ^1^Department of Epidemiology and Biostatistics, Tianjin Cancer Institute and Hospital, Tianjin 300060, China; ^2^Department of Physics, Tianjin University, Tianjin 300072, China

## Abstract

We have identified two genomic islands, that is, BCEGI-1 and BCEGI-2, in the genome of *Bacillus cereus* ATCC 10987, based on comparative analysis with *Bacillus cereus* ATCC 14579. Furthermore, by using the cumulative GC profile and performing homology searches between the two genomes, the integration sites of the two genomic islands were determined at single-nucleotide resolution. BCEGI-1 is integrated between 159705 bp and 198000 bp, whereas BCEGI-2 is integrated between the end of ORF BCE4594 and the start of the intergenic sequence immediately following BCE4626, that is, from 4256803 bp to 4285534 bp. BCEGI-1 harbors two bacterial Tn7 transposons, which have two sets of genes encoding TnsA, B, C, and D. It is generally believed that unlike the TnsABC+E pathway, the TnsABC+D pathway would only promote vertical transmission to daughter cells. The evidence presented in this paper, however, suggests a role of the TnsABC+D pathway in the horizontal transfer of some genomic islands.

## 1. Introduction

*Bacillus cereus* is a motile, spore-forming, and gram-positive bacterium, which is a soil-dwelling opportunistic pathogen causing both gastrointestinal and nongastrointestinal infections [1, 2]. The availability of the complete genome sequences of *Bacillus cereus* ATCC 14579 [3] and *Bacillus cereus* ATCC 10987 [4] provides an unprecedented opportunity to perform comparative analysis based on their genomes.

Genomic islands contain clusters of horizontally transferred genes [5, 6]. It is generally accepted that horizontal gene transfer (HGT) has an important role throughout the genome evolution in prokaryotes [7–21]. By transferring genes across species, HGT alters the genotype of a bacterium, which may lead to new traits, therefore, it has been described as “bacterial evolution in quantum leaps” [22].

Although the genome sequence of *B. cereus* ATCC 10987 is available [4], genomic islands of this genome have not been identified so far. Among the methods for detecting genomic islands, assessing the change in GC content remains an established way. The cumulative GC profile is a method that displays the distribution of GC content at a much higher resolution than that of the traditional window-based method [23]. Consequently, the method has been successfully used in identifying three genomic islands in the genome of *B. cereus* ATCC 14579 [24]. In this paper, the cumulative GC profile was used to identify two genomic islands in the genome of *B. cereus* ATCC 10987, based on comparative analysis with the genome of *B. cereus* ATCC 14579. Furthermore, based on an in-depth analysis of the homologous regions between the two genomes, we have determined the integration sites of the two genomic islands at single-nucleotide resolution.

## 2. Materials and Methods

The genome sequences of *B. cereus* ATCC 10987 and *B. cereus* ATCC 14579 were downloaded from the genome database at NCBI (http://www.ncbi.nlm.nih.gov/).

### 2.1. Using the Cumulative GC Profile to Calculate GC Content

We define

(1) $$\begin{array}{ll} z_{n} & =(A_{n}+T_{n})-(C_{n}+G_{n}),\\ n & =0,1,2,\ldots ,N,\;x_{n},y_{n},z_{n}\in [-N,N], \end{array}$$

where *A*_*n*_, *C*_*n*_, *G*_*n*_, and *T*_*n*_ are the cumulative numbers of the bases A, C, G, and T, respectively, occurring in the subsequence from the first base to the *n*th base in the DNA sequence inspected. *z*_*n*_ is one of the components of the Z curve, which is a three-dimensional curve that uniquely represents a DNA sequence [25, 26]. Usually, for an AT-rich (GC-rich) genome, *z*_*n*_ is approximately a monotonously increasing (decreasing) linear function of *n*. To amplify the deviations of *z*_*n*_, the curve of *z*_*n*_ ∼ *n* is fitted by a straight line using the least-square technique

(2) z=kn,

where (*z, n*) is the coordinate of a point on the straight line fitted and *k* is its slope. Instead of using the curve of *z*_*n*_ ∼ *n*, we will use the *z*′ curve, or cumulative GC profile, hereafter, where

(3) $$z_{n}^{\prime }=z_{n}-kn.$$

Let $\bar{\text{GC}}$ denote the average GC content within a region Δ*n* in a sequence, we find from (1), (2), and (3) that

(4) $$\overset{¯}{\text{GC}}=\frac{1}{2}(1-k-\frac{\Delta z_{n}^{\prime }}{\Delta n})\equiv \frac{1}{2}(1-k-k^{\prime }),$$

where *k*′ = Δ*z*′_*n*_/Δ*n* is the average slope of the *z*′ curve within the region Δ*n*. The region Δ*n* is usually chosen to be a fragment of a natural DNA sequence, for example, a genomic island. The above method is called the windowless technique for the GC content computation [23]. A program to draw the cumulative GC profile online is accessible from http://tubic.tju.edu.cn/zcurve/.

## 3. Results and Discussion

The cumulative GC profile is not the GC content itself. Rather, the derivative of the cumulative GC profile with respect to the base position *n* is negatively proportional to the GC content at the given position, that is, GC ∝ −*d* *z*′ /*dn* Therefore, the average slope of the cumulative GC profile within a region reflects the average GC content of the sequence within this region. An up jump in the cumulative GC profile indicates a relatively sharp decrease of GC content, whereas a drop indicates a relatively sharp increase of GC content.

**Figure 1:**
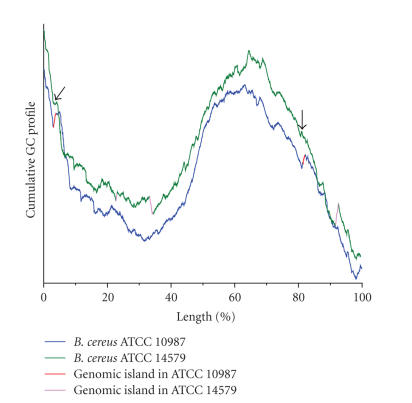
The cumulative GC profile for the genomes of *B. cereus* ATCC 10987 and *B. cereus* ATCC 14579. An up jump in the cumulative GC profile indicates a sharp decrease in GC content. By comparing the cumulative GC profiles for the two closed related genomes, it is shown that most parts of the two genomes overlap, whereas two jumps (marked in red) occur in the cumulative GC profile for the genomes of *B. cereus* ATCC 10987, suggesting that these two regions have a relatively sharp decrease in GC content. In addition, genomic sequences surrounding these two regions are highly conserved between the two genomes. Furthermore, these two regions also have other genomic-island specific features, such as the presence of Tn7 transposon. These lines of evidence suggest that the two regions are horizontally transferred genomic islands. Refer to text for detail. In the cumulative GC profile of the genome of *B. cereus* ATCC 14579, the regions that correspond to the integration sites of genomic islands are indicated by arrows.

**Figure 2:**
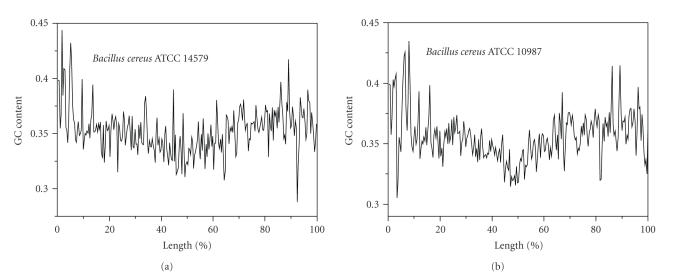
The GC content distribution computed based on 20 Kb windows sliding along the genomes of *B. cereus* ATCC 10987 and *B. cereus* ATCC 14579. Note that due to low resolution, the change in GC content, and the precise position of the change cannot be detected. Refer to Figure 1 for a comparison.

The cumulative GC profiles for the genomes of *B. cereus* ATCC 10987 and *B. cereus* ATCC 14579 show a similar pattern (Figure 1), suggesting that the two strains overall have a similar distribution of GC content along the genome. Three jumps are present in the genome of *B. cereus* ATCC 14579, and these three jumps correspond to three previously identified genomic islands [24]. Interestingly, there are also two jumps in the cumulative GC profiles of the *B. cereus* ATCC 10987 genome, indicating that the regions corresponding to these two jumps have a sharp decrease of GC content. In addition, the regions associated with these two jumps are absent in the *B. cereus* ATCC 14579 genome. Comparative analysis of the two *B. cereus* genomes exemplifies the high sensitivity of the cumulative GC profile. For instance, the traditional way to display the GC content distribution is to compute the GC content within a window that slides along the genome. However, using the window-based method, the detailed difference of the GC content distribution between the two *B. cereus* genomes, especially, the exact boundaries of regions showing the GC content difference, cannot be revealed due to the low sensitivity (Figure 2).

**Figure 3:**
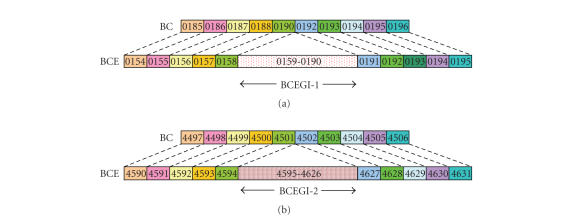
The regions that surround the genomic island BCEGI-1 and BCEGI-2 in the *B. cereus* ATCC 10987 genome and the corresponding regions in the genome of *B. cereus* ATCC 14579 are highly conserved. The same color denotes the homologous ORF's. (a) Conservation of gene orders around BCEGI-1. Briefly, except ORF's BCE0913 and BC0914, all corresponding ORF's are homologous. (b) Conservation of gene orders around BCEGI-2. All corresponding ORF's are homologous. BC denotes *B. cereus* ATCC 14579, whereas BCE denotes *B. cereus* ATCC 10987. The figure is not drawn to scale.

We also compared the genes that surround the regions corresponding to the up jumps in the cumulative GC profile of the *B. cereus* ATCC 10987. Consequently, we found that gene orders are highly conserved between the genome sequences surrounding the two regions and the corresponding regions in the *B. cereus* ATCC 14579 genome (Figure 3). Therefore, it is very likely that the two regions are horizontally transferred islands, which are designated the names BCEGI-1 and BCEGI-2, respectively.

In the *B. cereus* ATCC 10987 genome, the ORF's at the 5′ end of BCEGI-1, BCE0154, BCE0155, BCE0156, BCE0157, and BCE0158 are homologues of the ORF's in the *B. cereus* ATCC 14579 genome, BC0185, BC0186, BC0187, BC0188, and BC0190, respectively. At the 3′ end of BCEGI-1, the ORF's BCE0191, BCE0192, BCE0194, and BCE0195 are homologues of the ORF's BC0192, BC0193, BC0195, and BC0196, respectively (Figure 3).

The ORF's at the 5′ end of BCEGI-2, BCE4590, BCE4591, BCE4592, BCE4593, and BCE4594 are homologues of the ORF's in the *B. cereus* ATCC 14579 genome, BC4497, BC4498, BC4499, BC4500, and BC44501, respectively. At the 3′ end of BCEGI-2, the ORF's BCE4627, BCE4628, BCE4629, BCE4630, and BCE4631 are homologues of the ORF's BC4502, BC4503, BC4504, BC4505, and BC4506, respectively (Figure 3(b)).

**Figure 4:**
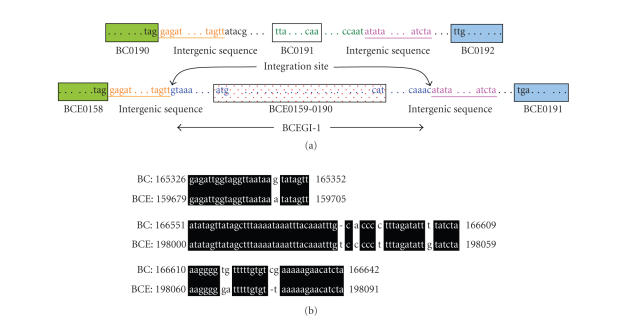
Determination of the integration sites of BCEGI-1 based on comparative analysis between *B. cereus* ATCC 10987 and *B. cereus* ATCC 14579. Besides gene orders, the intergenic sequences of the two genomes are also highly conserved. Therefore, the sequence segments that are absent in the genome of *B. cereus* ATCC 14579 are likely horizontally transferred. (a) Schematic diagram of BCEGI-1. The same color denotes homologous regions. The first or last codons of ORF's are marked. Integration sites are indicated. The figure is not drawn to scale. (b) Alignment of homologous intergenic sequences between the two genomes. BC denotes *B. cereus* ATCC 14579, whereas BCE denotes *B. cereus* ATCC 10987.

Therefore, it is highly likely that BCEGI-1 was integrated between the ORF's BCE0158 and BCE0191, whereas BCEGI-2 was integrated between the ORF's BCE4594 and BCE4627, respectively. Besides comparing at the gene level, we also performed homology searches at the sequence level to determine the exact integration sites. Indeed, sequences that flank BCEGI-1 are also homologous to some intergenic sequences in the *B. cereus* ATCC 14579 genome (Figure 4). An intergenic sequence adjacent to ORF BCE0158 is homologous to an intergenic sequence adjacent to ORF BC0190, whereas an intergenic sequence adjacent to ORF BCE0191 is homologous to an intergenic sequence adjacent to ORF BC0192 (Figure 4). Therefore, it is likely that BCEGI-1 is the segment of the genome between the sequences that have homologous counterparts in the *B. cereus* ATCC 14579 genome. According to this, BCEGI-1 starts at 159706 bp and ends at 197999 bp. Furthermore, it is likely that accompanying the integration of BCEGI-1, a gene that is homologous to BC0191, which encodes a membrane-spanning protein, was deleted from the *B. cereus* ATCC 10987 genome.

**Figure 5:**
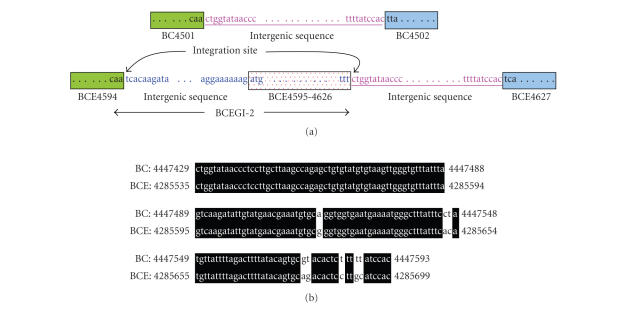
Determination of the integration sites of BCEGI-2 based on comparative analysis between *B. cereus* ATCC 10987 and *B. cereus* ATCC 14579. Besides gene orders, the intergenic sequences of the two genomes are also highly conserved. Therefore, the sequence segments that are absent in the genome of *B. cereus* ATCC 14579 are likely horizontally transferred. (a) Schematic diagram of BCEGI-2. The same color denotes homologous regions. The first or last codons of ORF's are marked. Integration sites are indicated. The figure is not drawn to scale. (b) Alignment of homologous intergenic sequences between the two genomes. BC denotes *B. cereus* ATCC 14579, whereas BCE denotes *B. cereus* ATCC 10987.

Likewise, sequences that flank BCEGI-2 are also homologous to sequences at the corresponding positions in the *B. cereus* ATCC 14579 genome. The intergenic sequence that is between the ORF's BCE4626 and BCE4627 in the *B. cereus* ATCC 10987 genome is homologous to the intergenic sequence between ORF's BC4501 and BC4502 in the *B. cereus* ATCC 14579 genome. The ORF BCE4594 is homologous to the ORF BC4501. Therefore, it is likely that BCEGI-2 was integrated between the end of ORF BCE4594 and the start of the intergenic sequence immediately following BCE4626 from 4256803 bp to 4285534 bp (Figure 5). However, BCEGI-2 is strikingly different in terms of integration sites. BCEGI-1 integrated into an intergenic sequence, and such integration resulted in a deletion of a segment of the genome sequence. However, BCEGI-2 integrated at a site immediately following an ORF, and such integration did not result in any deletion. We believe the accurate integration of BCEGI-2 into a site immediately following an ORF is not by coincidence, and it is likely that the different integration behaviors of BCEGI-1 and BCEGI-2 reflect the different integration mechanisms of these two horizontally transferred genomic islands.

BCEGI-1 is 38294 bp in length, with a GC content of 31.0%, whereas BCEGI-2 is 28732 bp in length, with a GC conent of 31.5%. The GC contents of both genomic islands are much lower than that of the genome, 35.6%.

BCEGI-1 contains 32 genes, which include two sets of Tn7 transposons. Transposons are DNA segments that can translocate from one place to another in the genome. The bacterial transposon Tn7 encodes an array of proteins that are involved in its transposition, that is, TnsA, B, C, D, and E [27]. In one set of Tn7 transposon in BCEGI-1, ORF's BCE0174, BCE0175, BCE0176, and BCE0177 encode Tn7-like transposition protein A, B, C, and D, respectively, whereas in the other set, ORF's BCE0182, BCE0183, BCE0184, and BCE0185 encode Tn7-like transposition protein A, B, C, and D, respectively. TnsA and TnsB together form the transposase that specifically recognizes the ends of the transposon. TnsC interacts with target DNA and TnsAB to promote the excision and insertion of Tn7. TnsD and TnsE are alternative target selectors, that is, Tn7 uses either the TnsABC+D or TnsABC+E to promote insertion by different mechanisms [27]. TnsABC+D mediated transposition specifically promotes transposition into a single chromosomal site, its attachment site or *attTn7*, which usually lies in the 3′ end of bacterial glutamine synthetase gene (*glms*) [28, 29]. No conserved *attTn7* sequence was found around BCEGI-1. However, BCEGI-1 is indeed at a location immediately following a *glms* gene (ORF BCE0158). It is generally believed that the TnsABC+E pathway would promote horizontal transfer between bacteria, whereas the TnsABC+D pathway would promote vertical transmission to daughter cells [27]. However, the evidence presented in this paper suggests an unexpected phenomenon, that is, the TnsABC+D pathway may promote the horizontal transfer of a genomic island.

BCEGI-2 contains 32 genes, including a *gerE* gene. *GerE* is a transcription factor that has been known to play an important role during the formation of spore, which protects the bacterium from adverse environmental conditions [30, 31]. *GerE* modulates the expression of some *cot* genes, which encode proteins that form the coat of mature spores [32, 33]. *B. cereus* is a spore-forming bacterium [1, 2]. Therefore, the presence of a *gerE* gene in a horizontally transferred genomic island suggests that HGT may play a role in the sporulation of *B. cereus*.

## 4. Conclusions

We have identified two genomic islands, that is, BCEGI-1 and BCEGI-2, in the genome of *B. cereus* ATCC 10987, based on comparative analysis with *B. cereus* ATCC 14579. Furthermore, by using the cumulative GC profile and performing homology searches between the two genomes, the integration sites of the two genomic islands were determined at single-nucleotide resolution. One genomic island harbors two bacterial Tn7 transposons, which have two sets of genes encoding TnsA, B, C, and D. It is generally believed that unlike the TnsABC+E pathway, the TnsABC+D pathway would only promote vertical transmission to daughter cells; the evidence presented in this paper, however, suggests a role of the TnsABC+D pathway in the horizontal transfer of some genomic islands.
